# Twenty Years-Passed Case of Demineralized Dentin Matrix Autograft for Sinus Bone Augmentation - A First Case of Dentin Graft in Human -

**DOI:** 10.4317/jced.60912

**Published:** 2023-10-01

**Authors:** Masaru Murata, Yukito Hirose, Morio Ochi, Junichi Tazaki, Naoto Okubo, Toshiyuki Akazawa

**Affiliations:** 1 Division of Regenerative Medicine, School of Dentistry, Health Sciences University of Hokkaido, Japan; 2 Division of Fixed Prosthodontics and Oral Implantology, School of Dentistry, Health Sciences University of Hokkaido, Japan; 3 Laboratory of Molecular and Cellular Medicine, Faculty of Pharmaceutical sciences, Hokkaido University, Sapporo, Japan; 4 Industrial Technology and Environment Research Development, Hokkaido Research Organization, Sapporo, Japan

## Abstract

This report presents a 20-year follow-up of a unique case involving a 46-year-old man who underwent sinus augmentation using autogenous demineralized dentin matrix (DDM) derived from non-functional teeth. Two extracted molars were crashed into granules, and then demineralized, freeze-dried, and stored at -80° for approximately one year. The stocked DDM granules were grafted into the sinus along with platelet-rich plasma, without the use of any membrane. Radiographic evidence at 1 month after the graft demonstrated successful harmonization of the augmented tissues with the atrophic maxilla, as shown by the increase in radiopaque dots. Computed tomography scans taken 5 months post-procedure revealed clear sinuses devoid of inflammation, significant bone formation, and a smooth buccal side outline. Bone biopsies at 5 months were carried out from the implant sites, and three fixtures were placed into the augmented bone. The biopsy tissues confirmed the presence of continuous trabecular bone linked with DDM, with new bone formation observed on it. A comparison of the dental X-ray images taken in 2009 and those captured in 2021 indicated minimal change in the outline of the new bone formed near the fixture-necks through the DDM graft and successful placement of dental implants was achieved. Based on this long-term case study, it is suggested that autogenous DDM graft could serve as a minimally invasive alternative for sinus bone augmentation without invasive bone harvesting and the associated morbidities.

** Key words:**Atrophic maxilla, autograft, bone, dentin, demineralized dentin matrix, sinus augmentation, teeth.

## Introduction

The atrophic posterior maxilla presents significant challenges when it comes to oral rehabilitation using implants. Techniques for sinus floor elevation and bone augmentation were first introduced in 1980 (1). Since then, lateral sinus floor elevation has become a standard surgical procedure for bone augmentation. A variety of materials have been employed for this purpose, including autogenous bone (1,2), allogenic bone (3), synthetic materials (4,5), recombinant human bone morphogenetic protein-2 delivered on a collagen sponge (BMP-2/collagen) (6), and autogenous blood-derived materials (7,8). Clinical studies on the BMP-2/collagen device have shown successful bone augmentation, as well as short-term safety and technical feasibility within 16 weeks (6). Blood-derived materials which contain thick fibrin fibers with platelets have also been explored (7,8). Animal studies have suggested that the demineralized dentin matrix (DDM) possesses bone-inductive abilities (9,10). This led to the first reported human DDM autograft for maxillary sinus bone augmentation in 2002, which was presented at the 81st International Association for Dental Research (IADR) conference in 2003. The surgical procedure for the initial DDM case involved lateral sinus floor elevation using a novel bone management technique with an ultrasonic scalar tip.

In this study, we focused on non-functional molars from a 48-year-old man as autogenous graft materials to avoid donor site morbidity. Two extracted molars were crushed and prepared as DDM granules, which was then recycled for sinus bone augmentation in the atrophic posterior maxilla in 2002 as part of a clinical study. The objectives of this report were to analyze the tissue biopsy from the implant sites taken 5 months post-sinus floor elevation and to assess the augmented tissues radiologically over a 20-year follow-up period.

## Case Report

-Patient

A 46-year-old male with an unremarkable medical history, bruxism, and heavy smoking habits (30 cigarettes/day) presented with multiple missing teeth and a broken bridge in 2000. He sought implant-based oral rehabilitation, having never used a partial denture. Initial X-rays and CT scans showed an atrophic jaw, impacted wisdom tooth with cyst-like lesion. The atrophic upper left jaw’s bone height measured 2.4-3.3 mm via CT.

-Surgery

**Figure 1:**
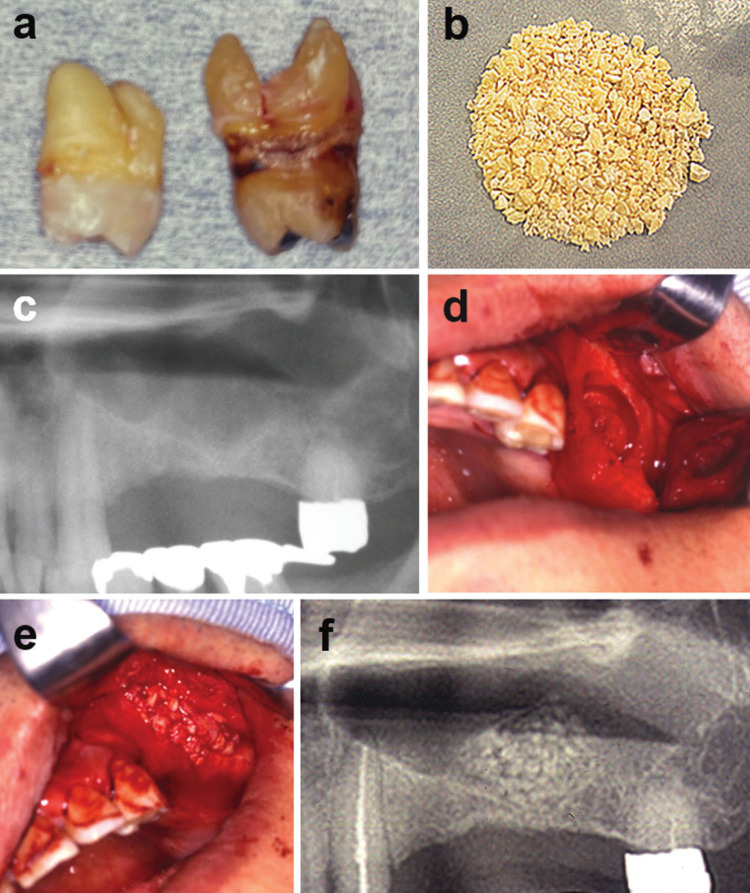
a,b: Preparations of partially demineralized dentin matrix (DDM). a. extracted molars (tooth #17, #18). b. freeze-dried DDM (total weight:2.06g). c-f: DDM autograft for sinus lifting in 48-year-old man. c. X-ray photo before sinus lifting. Note: 3 missing teeth (tooth #24,#25,#26) and atrophic maxilla. d. oval-shaped bone window. e. autogenous DDM graft. f. X-ray photos at 1 day after DDM graft. Ununiform of radio-opacity by partially demineralized dentin granules.

In 2000, a tooth (Tooth #25) associated with a broken bridge and an impacted tooth (Tooth #28) with a dentigerous cyst-like lesion were extracted. Non-functional, vital molars (Tooth #17, #18) were extracted in 2001 (Fig. 1a) and stored at -80°. These extracted molars were then cooled with liquid nitrogen and crushed in a stainless-steel vessel. The tooth granules were decalcified in a 0.6N HCl solution for 15 hours, creating DDM granules (Fig. 1b). These granules were rinsed in cold distilled water, freeze-dried, sieved to a size of 0.5mm to 2.0mm, and stored at -80° for future use. In May 2002, a sinus lifting procedure was performed using the stocked DDM. Panoramic X-ray photos were taken prior to the surgery (Fig. 1c). A bony window was then created using a fissure bur, and the bony surface was subsequently scratched with an ultrasonic scaler tip. The sinus membrane was then gently elevated with the scratched window bone (Fig. 1d). The DDM, mixed with 1.5 mL of platelet-rich plasma (PRP), was grafted into the sinus (Fig. 1e). The mucous-periosteal flap was repositioned without any membrane placement and sutured. Panoramic X-ray photos (Fig. 1f) and CT images were taken sequentially from the day after the sinus lifting procedure.

**Figure 2:**
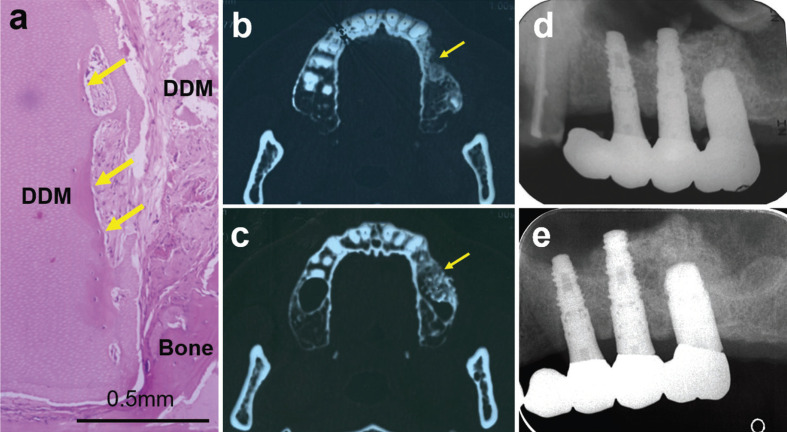
a. Biopsy tissues and histological views taken five months after sinus lifting. The arrows in the biopsy tissue indicate where the DDM granule has integrated with the bone and has been partially replaced by bone. b-e: CT photos at 5 months after sinus augmentation. b: ridge area of atrophic maxilla. Irregularly absorbed surface of buccal bone. c: Lower area of the DDM graft with smooth outlines on the buccal side (indicated by arrows). d-e: Comparison of dental X-ray images from 7 years and 19 years post-operation. d. X-ray photo in 2009 at 7 years after sinus augmentation. f. X-ray photo in 2021 at 19 years after sinus augmentation. The shadow of healthy bone near the fixture neck can be seen. In figures f and g, similarity of upper outlines of augmented bone.

Five months post-sinus lifting, implant surgery was planned. Upon flap elevation, it was observed that the DDM granules near the window had integrated with the new bone and were difficult to remove with an explorer. A bone biopsy was then performed at the implant sites (at tooth #26) for tissue observation (Fig. 2a), followed by the insertion of three fixtures into the augmented bone.

## Results

-Gross Views

The grafted DDM granules remained unexposed for 5 months following the sinus graft. The patient experienced no complications during 8-year follow-up period until 2009. Since then, he has received treatments in a private clinic near his workplace, and our University team involved with sinus bone augmentation had no contact with him for 14 years. Oral reports in 2022 from the private clinic indicated a deep bite similar to the initial presentation in 2000.

-Histological Findings of Tissue Biopsy obtained from Fixture Placements

The surface of the DDM bulk had been absorbed, giving it an irregular outline with lacunae. The DDM was seen to be harmonizing with the surrounding tissues and forming a connection with the bone (Fig. 2a). Cartilage was not observed in any of the specimens.

-Continuity of Radiographic Evaluations: Transition from Our Clinic to a Private Practice

Radiographic evaluations were conducted at various stages following the DDM graft, with images captured in our clinic from 2000 to 2009, and subsequently at a private clinic from 2009 onward. One day post-surgery, granular-shaped DDM materials were discernible between the elevated bone window plate and the atrophic maxilla. The dome-shaped augmentation, which included the DDM, reached a maximum height of approximately 15 mm. At 5 months, concave buccal lines were observed in the non-transplanted maxillary region (Fig. 2b), specifically at sites tooth#24 and tooth#25, as evident in the CT images. In contrast, within the DDM region, flat buccal lines were visible on the front wall of the left sinus, and a smooth outline was apparent on the buccal side of the augmented bone tissues (Fig. 2c), as revealed in the CT images. A comparison of the dental X-ray images taken at our clinic in 2009 (7 years post-operation; Fig. 2d) and those captured at a private clinic in 2021 (19 years post-operation; Fig. 2e) indicated minimal change in the outline of the new bone formed through the DDM transplantation. Finally, intraoral views and comprehensive dental X-ray images taken 20 years post-sinus augmentation highlighted the success of the bone augmentation for the dental implants. No instances of sinus inflammation were reported up until 2022.

## Discussion

**Figure 3:**
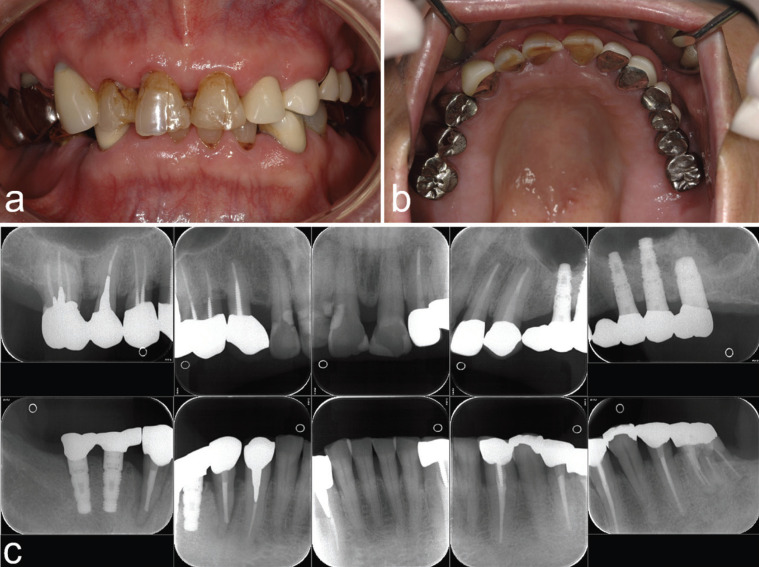
Intraoral views and dental X-ray photos in 2022 at 20 years after sinus augmentation. a: deep bite. b: view of maxilla. c: whole view of dental X-ray photos.

This clinical report describes a 20-year follow-up case of autograft using DDM granules for sinus augmentation in a 46-year-old man with severe atrophic posterior maxilla. Remarkably, this case was the world’s first autologous DDM in 2002. It stands out among global cases of autologous DDM grafts, as it has the longest follow-up period. Recent intraoral views and comprehensive dental X-ray images from 2022 reveal successful bone augmentation for oral rehabilitation (Fig. 3a-c). These long-term results provide evidences of the safety and technical feasibility of DDM augmentation.

-Bone Managements for Lateral Bone Window and Maxilla

The lateral bone window can either be elevated inward with the sinus membrane, crushed to produce bone chips, or repositioned back to its original location using the bone lid technique. In this case, we opted for the lateral bone-elevating method and grafted DDM, which acted as bone-inductive granules, into the three-dimensional space between the upwardly-elevated bone and the maxilla. To stimulate bone formation in the sinus, a scratching technique was employed using an ultrasonic scaler tip on the bony window. The scratched bone was elevated along with the sinus membrane, and then DDM granules were grafted into the newly created compartment. Our bone management techniques aimed at increasing the bone surface area. Consequently, we believe that DDM could act as bone-inductive collagenous material combined with PRP in the osteogenic space flanked by the upper and lower scratched bone. Even without a barrier membrane, the smooth buccal lines seen in the CT images at 5 months seem to indicate the regeneration of the periosteum and frontal bone (Fig. 2b,c). Additionally, a comparison of the dental X-ray images taken in 2009 and 2021 confirmed that the heights of the regenerated bone were almost same. This demonstrates the long-term stability of the bone regeneration achieved through DDM (Fig. 2d,e). Recent studies have shown that rat cortical bone that has been scratched has superior bone induction and conduction performance compared to unscratched cortical bone in subcutaneous tissues (11). Surprisingly, the scratched cortical bone induced bone formation as early as 2 weeks, while the non-scratched bone did not induce bone until 8 weeks (11). These results lend support to our case, suggesting that DDM quickly harmonized with the bone scratched by the ultrasonic scaler, enhancing the performance of sinus bone augmentation.

2. Biological Properties of Dentin and DDM

Natural dentin shares a biological similarity with cortical bone in terms of its components (12). Vital tooth-derived dentin constitutes an extracellular matrix abundant with growth factors and devoid of cells. Studies have shown that DDM performs better in bone regeneration compared to calcified dentin (11-14). The primary advantages of using partially demineralized dentin matrix materials are as follows: i) there’s no need to sacrifice a healthy donor site; ii) it contains growth factors essential for bone regeneration; iii) it has cross-linked collagen; iv) there’s no risk of immune rejection; v) it’s absorbable; vi) it contains the Arg-Gly-Asp (RGD) sequence, which promotes cell adhesion; vii) it’s hydrophilic; and viii) it retains mineral residues and mineral-binding proteins. However, one of the limitations is the volume constraint of DDM. We are of the view that the application of acellular DDM represents a pragmatic matrix-based therapy, as opposed to cell-based therapies that require significant costs and a lengthy period for cell culture in specialized cell-processing centers.

## Conclusions

The pioneering augmentation using autogenous DDM, first initiated in 2002, has been under radiological observation for 20 years. The biological application of vital molar-derived DDM has shown significant contribution to sinus bone augmentation for dental implant placements. From histological and radiological points of view, DDM granules have been observed to integrate well with the host and gradually be replaced by bone without causing sinus inflammation. This suggests that DDM autografting could be a feasible and minimally invasive procedure for patients.
